# Use of a FluoroType^®^ System for the Rapid Detection of Patients with Multidrug-Resistant Tuberculosis—State of the Art Case Presentations

**DOI:** 10.3390/diagnostics12030711

**Published:** 2022-03-15

**Authors:** Anna Zabost, Dorota Filipczak, Włodzimierz Kupis, Monika Szturmowicz, Łukasz Olendrzyński, Agnieszka Winiarska, Jacek Jagodziński, Ewa Augustynowicz-Kopeć

**Affiliations:** 1 Department of Microbiology, National Tuberculosis and Lung Diseases Research Institute, 01-138 Warsaw, Poland; d.filipczak@igichp.edu.pl (D.F.); e.kopec@igichp.edu.pl (E.A.-K.); 2 Department of Thoracic Surgery, National Tuberculosis and Lung Diseases Research Institute, 01-138 Warsaw, Poland; w.kupis@igichp.edu.pl; 3 1st Department of Lung Diseases, National Tuberculosis and Lung Diseases Research Institute, 01-138 Warsaw, Poland; monika.szturmowicz@gmail.com; 4 STOCER Mazovian Rehabilitation Center, Dr. Włodzimierz Roefler Memorial Hospital of the Polish Railroads, 05-800 Pruszków, Poland; l.olendrzynski@szpk.pl; 5 Department of Radiology, National Tuberculosis and Lung Diseases Research Institute, 01-138 Warsaw, Poland; a.winiarska@igichp.edu.pl; 6 X Department of Lung Diseases and Tuberculosis, Mazovian Centre of Lung Diseases and Tuberculosis, 05-400 Otwock, Poland; jjagodzinski@otwock-szpital.pl

**Keywords:** FluoroType MTBDR, tuberculosis, drug resistance, molecular, *Mycobacterium tuberculosis*, isoniazid, rifampin

## Abstract

According to the World Health Organization (WHO), there were 465,000 cases of tuberculosis caused by strains resistant to at least two first-line anti-tuberculosis drugs: rifampicin and isoniazid (MDR-TB). In light of the growing problem of drug resistance in *Mycobacterium tuberculosis* across laboratories worldwide, the rapid identification of drug-resistant strains of the *Mycobacterium tuberculosis* complex poses the greatest challenge. Progress in molecular biology and the development of nucleic acid amplification assays have paved the way for improvements to methods for the direct detection of *Mycobacterium tuberculosis* in specimens from patients. This paper presents two cases that illustrate the implementation of molecular tools in the recognition of drug-resistant tuberculosis.

## 1. Introduction

Tuberculosis, despite the considerable effort aimed at controlling its global spread, remains a public health challenge worldwide. One-third of the human population is currently estimated to harbor a *Mycobacterium tuberculosis* infection. According to the World Health Organization (WHO), there were approximately 10 million tuberculosis cases in 2019, 465,000 of which were cases of tuberculosis caused by strains resistant to at least two first-line anti-tuberculosis drugs: rifampicin and isoniazid (MDR-TB) [1]. The most important reasons for the increasingly deteriorating epidemiological situation of tuberculosis worldwide are: poor results of tuberculosis control programs and insufficient implementation thereof, lack of funds for treatment in developing countries, the spread of HIV infection, and drug resistance in *Mycobacterium tuberculosis*, which is considered by WHO experts to be a major driver of tuberculosis in the modern world.

The WHO definition of a tuberculosis case requires microbiological confirmation of the disease, which involves the isolation of the causative agent, namely bacteria belonging to the *Mycobacterium tuberculosis* complex (MTC), determination of the species, and drug susceptibility testing. The monitoring of TB programs is aimed at the rapid identification of patients, prompt initiation of appropriate treatment, and monitoring of the treatment progress, thus preventing further transmission of *Mycobacterium tuberculosis*. According to statistical data, one patient with tuberculosis infects about 15 individuals annually.

The achievement of the gold standard in the diagnosis of tuberculosis, which consists of microbiological confirmation in a culture, is a difficult process due to the long generation time for the genus Mycobacterium of about 18 h. Due to the slow growth of mycobacteria on bacteriological culture media, *Mycobacterium tuberculosis* infection is diagnosed after several days in the case of liquid media (Bactec MGIT; Becton Dickinson) or several weeks in the case of solid media (Löwensteina-Jensena (LJ)) [2].

Progress in molecular biology and the development of nucleic acid amplification assays have paved the way for improvements to methods for the direct detection of *Mycobacterium tuberculosis* in specimens from patients. These techniques make it possible to detect and identify the *Mycobacterium tuberculosis* complex at a higher sensitivity and within a shorter period of time compared to conventional methods, which is particularly important in cases that are difficult to diagnose. This mainly applies to the paucibacillary extrapulmonary form of tuberculosis.

In light of the growing problem of drug resistance in *Mycobacterium tuberculosis* across laboratories worldwide, the rapid identification of drug-resistant strains of the *Mycobacterium tuberculosis* complex poses the greatest challenge. Early detection and diagnosis of drug resistance make it possible to initiate an appropriate treatment regimen. The introduction of genetic tests in routine diagnostic procedures enables the quick detection of resistance to rifampicin due to the identification of a mutation in the *rpoB* gene. The implementation of FluoroType^®^ MTBDR VER. 2.0 (Bruker) additionally allows us to define mutations in the *katG* and *inhA* genes that determine resistance to isoniazid. According to WHO and ECDC (European Centre for Disease Prevention and Control) recommendations, the progressive increase in the prevalence of drug-resistant tuberculosis requires accurate and rapid diagnostic tools [2].

The presented case series illustrates the implementation of such tools in the recognition of drug-resistant tuberculosis. Case 1 concerns the prompt molecular diagnosis of *M. tuberculosis* strains monoresistant to isoniazid directly from surgical lung specimens. Case 2 illustrates the possibility of the quick molecular diagnosis of MDR-TB from sputum.

## 2. Case Presentation

### 2.1. Case 1

**Figure 1:**
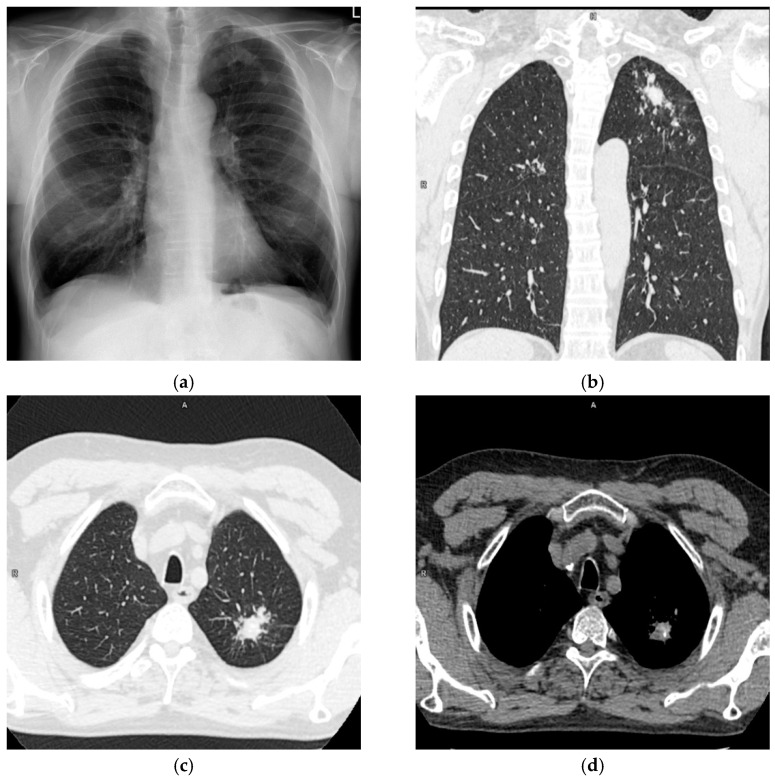
Chest X-ray and CT (**a**). Chest X-ray, posteroanterior projection. Irregular shape consolidation in the upper zone of the left lung. (**b**). Chest CT, lung window, coronal image. Various shape and size lung nodules in the left upper lobe. (**c**) Chest CT, lung window, axial image. The largest lung nodule with spiculated borders is in the left upper lobe. (**d**) Chest CT, mediastinal window, axial image. Calcifications in lung nodules.

A 62-year-old male was admitted to the Department of Thoracic Surgery of National Tuberculosis and Lung Diseases Research Institute due to a focal lesion localized in the left lung. Irregular shape consolidation in the upper zone of the left lung was found in a chest X-ray (Figure 1a). Chest CT (chest computed tomography) revealed several nodules of various shapes and sizes, with small calcifications, localized in the apicoposterior segment of the left lung. The largest nodule, measuring 28 × 23 mm, had spiculated borders (Figure 1b–d). These findings were ambiguous, requiring differentiation between tuberculosis and neoplasm.

Bronchoscopy was unremarkable. Open surgical biopsy revealed several pulmonary nodules, 10–12 mm in size, localized in segment two of the left lung. The intraoperative pathological examination documented the presence of an inflammatory lesion with signs of necrosis. No neoplastic cells were found. Subsequently, left segmentectomy 1 + 2 + 3 was performed, with lymphadenectomy in groups 5, 7, and 11.

A lung fragment was collected to test for tuberculosis and non-tuberculous mycobacterial infections. Smear microscopy revealed acid-fast bacilli. The patient was in contact with a person suffering from tuberculosis. In order to determine the mycobacterial species, genetic testing with the GeneXpert MTB/RIF (Cepheid) system was carried out, which confirmed the presence of a *Mycobacterium tuberculosis* complex susceptible to rifampicin in the specimen tested. The same clinical specimen was used for molecular testing with the FluoroType^®^ system. The test confirmed the presence of the genetic material of *Mycobacterium tuberculosis* and identified, at the same time, the presence of the S315T1 mutation in the *katG* gene, which confers the resistance to isoniazid (INH). Neither the FluoroType^®^ system nor the GeneXpert^®^ system identified any mutations responsible for resistance to rifampicin.

On histopathology, multiple necrotizing granulomas were found. Ziehl-Neelsen’s staining for mycobacteria was positive. Resected lymph nodes showed signs of pneumoconiosis and a few non-necrotizing granulomas.

After 17 days, the liquid culture became positive for *Mycobacterium tuberculosis*, and the result was confirmed by a test that detects the production of the MPT64 protein. The strain grown in the culture was subjected to a drug susceptibility test using the Bactec MGIT system and molecular identification of drug resistance using the GenoType MTBDRplus assay (Bruker). Genetic testing confirmed the S315T1 mutation in the *katG* gene, previously detected using the FluoroType^®^ system. A phenotypic assay for drug resistance confirmed that the strain was resistant to isoniazid only.

The patient was transferred to a tuberculosis inpatient department for tuberculosis treatment.

### 2.2. Case 2

A 41-year-old female, emaciated, with alcohol dependence syndrome, treated for sensitive tuberculosis in 2016, was admitted to hospital due to productive cough, progressive general weakness, shortness of breath, and difficulty in swallowing foods and liquids.

**Figure 2:**
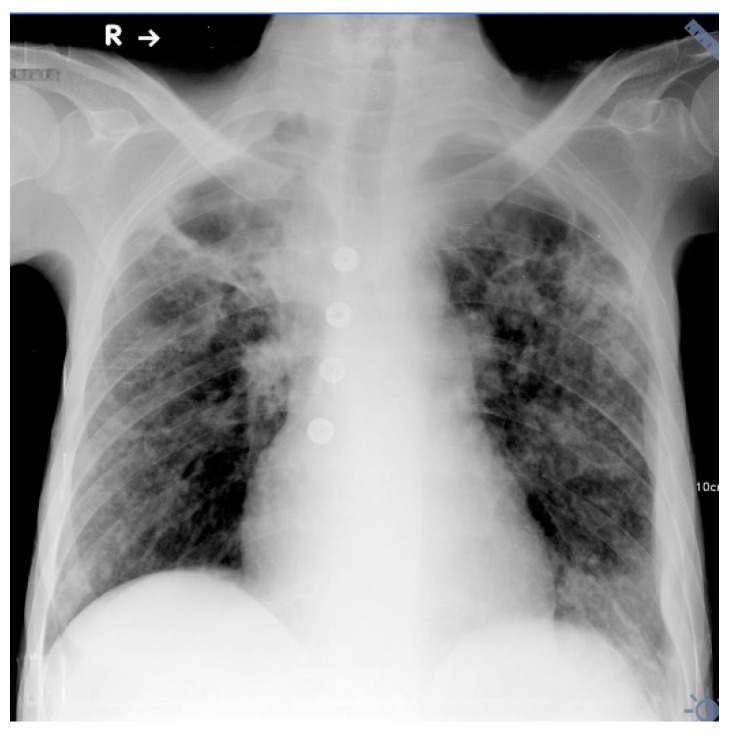
Chest X-ray anteroposterior in a horizontal position. Extensive, parenchymal consolidations in both lungs with low-attenuation areas in upper zones suggest cavitations.

The chest radiograph showed extensive, parenchymal consolidations in both lungs, with low-attenuation areas in upper zones suggesting cavitations (Figure 2).

Laboratory tests revealed increased levels of CRP (C reactive protein), INR (international normalized ratio) and aminotransferases, and decreased levels of total protein, albumin, iron, folic acid, and calcium. As part of the workup, sputum was collected to test for tuberculosis and non-tuberculous mycobacterial infections. Smear microscopy revealed a very high count of acid-fast bacilli. Genetic testing with the GeneXpert MTB/RIF revealed the presence of a *Mycobacterium tuberculosis* complex and identified resistance to rifampicin (RMP). As the patient was suspected of having multidrug-resistant tuberculosis, molecular testing was also carried out with the FluoroType^®^ system. The test confirmed the presence of the genetic material of *Mycobacterium tuberculosis*, the S531L mutation in the *rpoB* gene, and the S315T1 mutation in the *katG* gene, allowing us to identify multidrug resistance (MDR). After six days, a positive culture on the MGIT liquid medium was obtained, and a test that detects the production of MPT64 protein was carried out, which confirmed that the identified bacteria belonged to the *Mycobacterium tuberculosis* species. The GenoType molecular drug resistance assay confirmed the presence of the S531L mutation in the *rpoB* gene and the S315T1 mutation in the *katG* gene, allowing us to identify multidrug resistance. Further analysis of drug resistance in the grown strain, using the GenoType MTBDRsl assay, allowed us to classify the strain as an extensively drug-resistant (XDR) strain. A molecular analysis based on spoligotyping qualified the strain to the Beijing 1 molecular family. The patient was admitted to the tuberculosis inpatients department to start appropriate therapy; nevertheless, she died 10 days later.

## 3. Discussion

The fundamental and best method of preventing tuberculosis is based on breaking the mycobacterial transmission chains in the population. Early detection of sputum-positive patients requires appropriate organization of medical services and the correct use of the available diagnostic methods. Patients who shed mycobacteria that are resistant to first-line drugs pose an obstacle to the rapid and effective eradication of tuberculosis. The risk of transmission increases with multidrug-resistant tuberculosis, as the period of patients’ infectivity is prolonged in these cases. The rates of primary and acquired drug resistance and the rates of MDR strains are a measure of the quality of tuberculosis surveillance in a given country. It is estimated that 3.3% of new patients and 18.0% of previously treated patients worldwide are individuals with MDR tuberculosis. The largest number of cases of MDR tuberculosis is recorded in Eastern Europe and Central Asia, where they account for over 20% of new patients and over 50% of previously treated patients [1]. In Poland, according to the Central Registry of Tuberculosis, 39 patients with MDR tuberculosis were registered in 2019, accounting for 1.0% of all bacteriologically confirmed tuberculosis cases [3].

Analysis of the prevalence of drug-resistant tuberculosis helps in the detection and monitoring of the spread of MDR and XDR strains and illustrates the effectiveness of tuberculosis surveillance in a given country. Multidrug-resistant tuberculosis is more difficult to cure than drug-susceptible tuberculosis; it requires longer, two-year treatment, and permanent conversion is achieved in fewer than 50% of new patients and in 30% of previously treated patients. In the course of a correct workup, rapid detection of *Mycobacterium tuberculosis* is important. This is currently possible through the use of genetic methods, which are able to identify patients with multidrug-resistant tuberculosis within a few hours of specimen collection. Early generations of the commercial tests used to detect *Mycobacterium tuberculosis* (ProbeTec ET DTB and COBAS TaqMan MTB) were characterized by relatively low sensitivities and directly detected the genetic material of *Mycobacterium tuberculosis* in patient specimens without providing information on drug resistance. The currently available kits, FluoroType^®^ MTBDR VER. 2.0 and Xpert^®^ MTB/RIF Ultra, are characterized by a much higher sensitivity and, at the same time, offer the possibility of rapidly identifying patients with drug-resistant tuberculosis (Table 1) [4,5].

Xpert^®^ MTB/RIF are closed, virtually fully automated semi-quantitative assays based on real-time PCR. They offer the possibility of the simultaneous detection of *Mycobacterium tuberculosis* DNA in specimens from patients with suspected tuberculosis, directly or after their processing, and the detection of mutations determining rifampicin resistance in the *rpoB* gene. This assay is able to provide a result for a single sample within just two hours [6]. Limitations of the GeneXpert^®^ MTB/RIF Ultra assay include the lack of the option to identify mono-resistance to isoniazid and the fact that resistance to rifampicin is not always a surrogate for MDR. Augustynowicz-Kopeć et al. demonstrated that, in the Polish population, resistance to rifampicin in the group of newly diagnosed patients is a surrogate marker of MDR tuberculosis in a mere 30% of the cases [7]. The situation is different in the group of previously treated patients, where the predictive precision is approximately 80%, and resistance to rifampicin may be a surrogate marker of MDR [7,8]. A meta-analysis published in 2019 demonstrated that INH mono-resistant tuberculosis was more common in younger patients than drug-resistant tuberculosis (median age: 41 vs. 46 years) and affected more foreigners (37.0% vs. 28.6%) and more previously treated patients (13.6% vs. 9.4%). Cases of INH resistant tuberculosis were more commonly reported in countries with high incidence rates for tuberculosis than those with lower incidence rates (65.9% vs. 60.0%). The prevalence of isoniazid mono-resistance ranges from 1.1% in Slovakia to 66.7% in Iceland [9]. The same analysis revealed that one year after treatment initiation, cases of INH mono-resistant tuberculosis had lower treatment success rates compared to cases with drug-susceptible tuberculosis (67.7% vs. 75.8%), which allowed the authors to hypothesize that INH mono-resistance negatively impacted the treatment outcomes. Therefore, the results of this analysis emphasize the need to identify patients with INH mono-resistant tuberculosis, especially in view of the fact that the rapid tests in recent years focused more on detecting resistance to rifampin as a surrogate marker of MDR tuberculosis [9,10].

A test that identifies patients with INH or RMP mono-resistance and patients with MDR-TB is FluoroType^®^MTBDR VER.2.0. The test enables rapid identification of *Mycobacterium tuberculosis* in clinical specimens and identification of the most common mutations associated with rifampicin and isoniazid resistance. The sensitivity and specificity of the FluoroType^®^ system for the detection of rifampicin resistance in *Mycobacterium tuberculosis* strains are comparable to those obtained with the GenoType MTBDRplus and GeneXpert^®^ systems [11,12]. The isoniazid resistance detection sensitivity with this system is 91.7% at a specificity of 100% [11]. In the first analysis of the usefulness of the FluoroType^®^ system in detecting the genetic material of *Mycobacterium tuberculosis* in clinical specimens, the sensitivity and specificity of the FluoroType^®^ system were shown to correlate with smear microscopy results, at 98% for AFB-positive specimens and 92% for AFB-negative specimens. At the same time, FluoroType^®^MTBDR VER.2.0 was shown to be characterized by high accuracy in detecting mutations in *rpoB*, *katG*, and *inhA*: 98%, 97%, and 97%, respectively. Such high accuracy in identifying mutations determining rifampicin and isoniazid resistance results in 100% sensitivity and specificity in detecting MDR-TB strains with the FluoroType^®^ system. One advantage of the FluoroType^®^ system is that 94 samples can be analyzed simultaneously within three hours and that the results are analyzed automatically, which removes the subjectivity of their interpretation [13].

In the cases presented here, the use of the FluoroType^®^ system enabled the rapid and correct identification of drug-resistant tuberculosis. In the first case, a microbiological workup of the 62-year-old male identified a strain of *Mycobacterium tuberculosis* mono-resistant to INH. In the second case of the 41-year-old female, the FluoroType^®^ system enabled the identification of MDR-TB directly from the specimen. In both cases, using only the GeneXpert system in the microbiological diagnostic algorithm for tuberculosis would not have provided the opportunity to correctly identify drug-resistant tuberculosis.

## 4. Conclusions

Rapid testing for tuberculosis, which includes the identification of resistance to tuberculosis drugs, is necessary to initiate effective treatment and halt disease transmission. A new generation of more sensitive, automated molecular diagnostic assays enables simultaneous detection of rifampicin and isoniazid resistance markers. An indubitable advantage of molecular assays is the short turnaround time and the possibility of starting the patient on the correct treatment. However, the results of numerous studies showed that drug resistance in *Mycobacterium tuberculosis* may be caused by other mutations in a region of the gene that is not analyzed by a given genetic test. Further research is therefore required to improve the molecular assays and to include new mutations responsible for resistance to tuberculosis drugs in commercial assays. Currently, in order to correctly determine the resistance of *Mycobacterium tuberculosis* strains to tuberculosis drugs, genetic testing must be supplemented with conventional drug resistance tests [14]. The presented case series illustrated the role of FluoroType^®^ MTBDR VER. 2.0 in diagnosing the M. tuberculosis strain with isoniazid monoresistance as well as MDR-TB, directly from clinical samples.

## Figures and Tables

**Table 1:** Comparison of genetic tests for the detection of *Mycobacterium tuberculosis* complex.

Molecular Test	Rifampicin Resistance	Isoniazid Resistance	Limit of Detection (CFU/mL)	Test Time (h)
Xpert^®^MTB/RIF ULTRA	*rpoB*	no	11	2
Fluorotype MTBDR VER.2.0	*rpoB*	*KatG inhA*	15	2.5

## Data Availability

The clinical data of the patients are available in the hospital database.

## References

[1] (2020). Global Tuberculosis Report 2020.

[2] European Centre for Disease Prevention and Control (2018). Handbook on Tuberculosis Laboratory Diagnostic Methods in the European Union–Updated 2018.

[3] Korzeniewska-Koseła M. (2020). Tuberculosis and Respiratory Tract Diseases in Poland in 2019.

[4] Antonenka U., Hofmann-Thiel S., Turaev L., Esenalieva A., Abdulloeva M., Sahalchyk E., Alnour T., Hoffmann H. (2013). Coparison of Xpert MTB/RIF with ProbeTec ET DTB and COBAS TaqMan MTB for direct detection of *M. tuberculosis* complex in respiratory specimens. BMC Infect. Dis..

[5] Hofmann-Thiel S., Hoffmann H. (2014). Evaluation of Fluorotype MTB for detection of *Mycobacterium tuberculosis* complex DNA in clinical specimens from a low-incidence country. BMC Infect. Dis..

[6] Albert H., Nathavitharana R.R., Isaacs C., Pai M., Denkinger C.M., Boehme C.C. (2016). Development, roll-out and impact of Xpert MTB/RIF for tuberculosis: What lessons have we learnt and how can we do better?. Eur. Respir. J..

[7] Augustynowicz-Kopeć E. (2007). Drug-Resistant Tuberculosis in Poland. An Epidemiological, Microbiological, and Genetic Analysis. Habilitation Thesis.

[8] Karo B., Kohlenberg A., Hollo V., Duarte R., Fiebig L., Jackson S., Kearns C., Ködmön C., Korzeniewska-Koseła M., Papaventsis D. (2018). Isoniazid mono-resistance negatively affects tuberculosis treatment outcomes in Europe. Eur. Respir. J..

[9] Karo B., Kohlenberg A., Hollo V., Duarte R., Fiebig L., Jackson S., Kearns C., Kodmon C., Korzeniewska-Koseła M., Papaventsis D. (2019). Isoniazid (INH) mono-resistance and tuberculosis (TB) treatment success: Analysis of European surveillance data, 2002 to 2014. Euro. Surveill..

[10] Stagg H.R., Lipman M.C., McHugh T.D., Jenkins H.E. (2017). Isoniazid-resistant tuberculosis: A cause for concern?. Int. J. Tuberc. Lung Dis..

[11] Hillemann D., Haasis C., Andres S., Behn T., Kranzer K. (2018). Validation of the FluoroType MTBDR Assay for Detection of Rifampin and Isoniazid Resistance in *Mycobacterium tuberculosis* complex isolates. J. Clin. Microbiol.

[12] Steingart K.R., Schiller I., Horne D.J., Pai M., Boehme C.C., Dendukuri N. (2014). Xpert(R) MTB/RIF assay for pulmonary tuberculosis and rifampicin resistance in adults. Cochrane Database Syst. Rev..

[13] de Vos M., Derendinger B., Dolby T., Simpson J., van Helden P.D., Rice J.E., Wangh L.J., Theron G., Warren R.M. (2018). Diagnostic accuracy and utility of FluoroType MTBDR, a new molecular assay for multidrug-resistant tuberculosis. J. Clin. Microbiol..

[14] Iacobino A., Fattorini L., Giannoni F. (2020). Drug-Resistant Tuberculosis 2020: Where we stand. Appl. Sci..

